# Cardiomyocyte-specific circulating cell-free methylated DNA in esophageal cancer patients treated with chemoradiation

**DOI:** 10.3390/gidisord3030011

**Published:** 2021-07-30

**Authors:** Sarah Martinez Roth, Eveline E. Vietsch, Megan E. Barefoot, Marcel O. Schmidt, Matthew D. Park, Archana Ramesh, Michael R. Lindberg, Giuseppe Giaccone, Anna T. Riegel, Ana Barac, Keith Unger, Anton Wellstein

**Affiliations:** Lombardi Comprehensive Cancer Center, Georgetown University, Washington, DC, USA

**Keywords:** esophageal cancer, therapy response, circulating cell-free DNA, DNA methylation, amplicon sequencing

## Abstract

Thoracic high dose radiation therapy (RT) for cancer has been associated with early and late cardiac toxicity. To assess altered rates of cardiomyocyte cell death due to RT we monitored changes in cardiomyocyte-specific, cell-free methylated DNA (cfDNA) shed into the circulation. Eleven patients with distal esophageal cancer treated with neoadjuvant chemoradiation to 50.4 Gy (RT) and concurrent carboplatin and paclitaxel were enrolled. Subjects underwent fasting blood draws prior to the initiation and after completion of RT as well as 4–6 months following RT. An island of six unmethylated CpGs in the FAM101A locus was used to identify cardiomyocyte-specific cfDNA in serum. After bisulfite treatment this specific cfDNA was quantified by amplicon sequencing at a depth of >35,000 reads/molecule. Cardiomyocyte-specific cfDNA was detectable before RT in the majority of patient samples and showed some distinct changes during the course of treatment and recovery. We propose that patient-specific cardiac damages in response to the treatment are indicated by these changes although co-morbidities may obscure treatment-specific events.

## 1. Introduction

The number of patients living with cancer and cancer survivors is increasing in the United States due to advances in early detection, improved cancer treatment and the aging population [1]. Development of novel therapies, often given in sequence or concomitantly with conventional therapies, has resulted in prolonged survival but also carries an increased risk of adverse events. Cardiovascular toxicities from conventional cytotoxic or pathway-targeted therapies, immunotherapy, radiation treatment have been recognized and can include myocardial infarction, left ventricular dysfunction, hypertension, heart failure, coronary artery disease and arrhythmias [1][2]. Here, we evaluate the use of circulating nucleic acids as a potential marker of cardiac injury related to cancer therapies and present feasibility data in esophageal cancer patients treated with chemoradiation.

Liquid biopsies or blood sample-based diagnostics in patients with cancer were originally focused on the harvest of circulating tumor cells (CTCs) to gain insights into the makeup of invasive and metastatic cancers that shed cells into the blood stream [3][4]. The impact of circulating biomarkers has grown as technological advances increase the sensitivity of molecular biology techniques and allow for non-invasive characterization of molecular determinants in cancer for real time monitoring of individual patients. Circulating cell-free DNA (cfDNA) has been shown in previous studies to distinguish disease in early and late stage cancers without requiring invasive procedures to obtains samples from primary or metastatic lesions [5][6][7]. More recent uses of blood sample analyses to gain insights into the molecular composition of cancers have expanded to cfDNA, mRNAs and microRNAs (miR) as diagnostic or prognostic biomarkers in cancer [4][8]. Earlier studies have shown that changes in the expression patterns of circulating miRNAs can be indicators for responsiveness to drugs [4][9]. cfDNA analysis can also detect the presence of cancer by monitoring the abundance of mutant DNA that is shed from dying cancer cells into the circulation. Numerous studies have shown that liquid biopsies can be used to accurately infer molecular characteristics and provide a comprehensive view of tumor genetics that encompasses multiple tumor subtypes. One of the more recent developments along these lines is the FDA approval of cfDNA analysis to alter treatment for drug resistant lung cancers upon detection of an indicator T790M EGFR mutation in cfDNA [10].

An epigenetic marker known as DNA methylation is erased in early metazoan organism development and reestablished over the lifetime of the organism [11]. DNA methylation has also been shown to be a useful tool to study cancer and aging [12]. Interestingly, fragments of cellular genomic DNA from normal cells are detectable in the bloodstream due to the physiologic turnover and replacement of cells in healthy organs at steady-state. Damage of healthy organs due to various insults can result in higher cellular turnover rates that are reflected in an increase in fragments of genomic DNA shed from dying cells. Previous work by others pioneered the detection of tissue-specific cell death based on the presence of tissue-specific methylation patterns in cfDNA [13][14]. This approach can be adapted to identify cfDNA from any cell type in the body [13] and was reviewed by us recently [15]. Complementary to methylation patterns in cfDNA nucleosome footprints can also be used to infer cell types contributing to the altered abundance of cfDNAs in different pathological states including cancer [16]. Quite diverse insults including hypoxia, trauma, immune attack, or exposure to toxic chemicals or drugs can be detected as changes in methylated cfDNA composition originating from different dying cell populations [17].

cfDNA is an attractive blood-based marker due to its relative stability in plasma and serum with a relatively short *in-vivo* half-life of up to 3 hours [18]. A proof of principle study tested the utility of circulating cell-free donor-derived DNA (dd-cfDNA) in monitoring acute rejection after heart transplantation in a small retrospective cohort [18]. In addition to using cfDNA as a diagnostic marker to detect acute rejection after heart transplantation, cfDNA has been shown to be a useful biomarker for cardiomyocyte death to monitor cardiac pathologies such as myocardial infarctions [19]. In other organs such as the liver, damage is usually assessed by serum measurements of enzymes such as aspartate aminotransferase (AST) and alanine aminotransferase (ALT). As shown recently, hepatocyte-derived cfDNA can provide information on hepatocyte death during disease and toxicity [20]. Other groups have shown that cfDNA can be used to detect organ-specific signatures that correlate with rejection of any combination of donors and recipients, and may be applicable to other solid organ transplants [21].

In the cancer setting, whole exome sequencing of cfDNA concordance has been used in monitoring metastatic disease and potentially discover patients with earlier stages of disease [22]. The ability to use cancer-specific altered DNA methylation in liquid biopsies can complement the prediction, monitoring, and diagnosis of cancer [23]. Circulating cell-free tumor DNA (ctDNA) has also been shown to uncover residual disease in patients with early stage colon cancer [24]. Serial analysis of ctDNA and circulating tumor cells (CTCs) can also be a useful, noninvasive tool to monitor clonal evolution during the progression of disease [25]. In conclusion, ctDNA has been shown to be a specific and sensitive biomarker that can be used to detect a variety of different tumor types [26], and has been used to detect mutations occurring at very low allele frequencies [27].

The tissue source of cfDNA fragments in the circulation can be delineated from the distinct patterns of DNA methylation that distinguish cells from different tissues [14]. DNA methylation is an epigenetic regulatory mechanism that is highly stable and cell-type specific. DNA methylation marks are erased in early metazoan organism development and reestablished over the lifetime of the organism as cell fate decisions are made. From a comparison of DNA methylation of tissues, genomic sequences can be selected which will allow for detection of tissue-specific DNA fragments in the circulation [28]. Thus, complementary to mutation analysis of cfDNA shed from cancerous cells, changes in DNA methylation patterns of cfDNA can reveal abnormal tissue homeostasis and altered rates of cell death in healthy normal tissues [29]. Newer studies have shown that deconvolution of cfDNA can be used to relate changes to clinical findings [30]. This makes DNA methylation an appealing tool where markers can be selected through identifying sites in reference data that are preferentially hyper- or hypo-methylated in specific tissues or cell types.

In the present proof of principle study, we evaluated whether radiation treatment related cardiac damage can be detected through changes in circulating tissue-specific DNA in cancer patients. We hypothesized that changes in cardiomyocyte-specific, methylated DNA in the circulation could be used to detect increased cardiac cell death in patients undergoing cancer treatment and potentially serve as an early marker of cardiac toxicity. Here, we describe the rationale and development of an amplicon sequencing method to detect changes in cardiac cfDNA methylation patterns in comparison to current clinical markers such as BNP, CRP, Troponin-1, and LVDEV. We present preliminary data from patients with esophageal cancer treated with radiation and chemotherapy.

## 2. Materials & Methods

### 2.1. Experimental Approach

**Figure 1.**
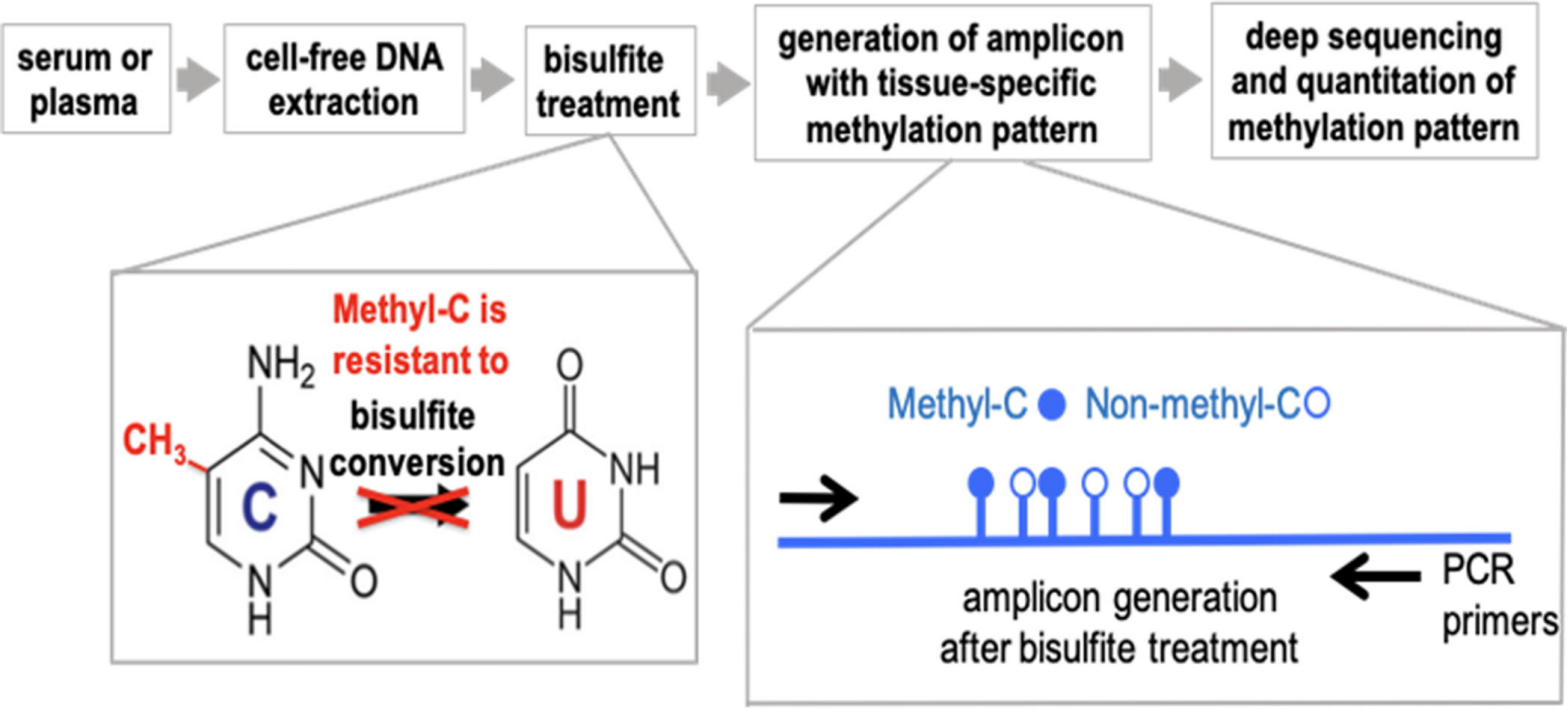
Overview of approach to detect to changes in circulating cell-free DNA (cfDNA) methylation patterns. Cell- free DNA is first extracted from plasma or serum, then cfDNA is bisulfite converted. Bisulfite conversion is the gold standard for methylation sequencing, where methylated cytosines remain cytosines in sequencing and bisulfite converted cytosines become uracils. Next, amplicons with tissue-specific methylation pattern as generated using PCR analysis. That is followed by deep sequencing and quantitation of methylation pattern.

The experimental paradigm is outlined in Figure 1. The approach to detect differentially methylated DNA takes advantage of the well-established distinct sensitivity between methylated and non-methylated cytosines to bisulfite treatment of DNA that converts non-methylated cytosines to uracil. The change is detected by conventional DNA sequencing where methylated cytosines will be read as Cs whereas non-methylated cytosines will be read as Ts. For the detection and quantitation of DNA fragments in the circulation we use a PCR-based approach that relies on Next Generation (next-gen) Sequencing of DNA amplicons to quantify sequence-specific methylation within the selected fragments. Non-methylation specific PCR primers were designed to hybridize outside of target CpG sites to generate the amplicons from bisulfite-treated DNA and quantify the number of methylated and non-methylated CpGs after next-gen deep sequencing.

### 2.2. Genomic DNA from different Organs

Commercially available human genomic DNA from different organs was used to establish tissue specificity. The DNA sources were cardiac myocytes (ScienCell cat. #6219), heart left ventricle (Amsbio cat. #D1234138), skeletal muscle (Amsbio cat. #D1234171), lung (Amsbio cat. #HG-601), spleen (Amsbio cat. # CD563320). We also included universal methylated human DNA standard (Zymo cat. #D5011).

### 2.3. Normal Healthy Donors

We acquired serum from three normal controls from the histopathology and tissue shared resource at Georgetown Lombardi Cancer Center. Subjects included: 36604 (age 25, female), 32519 (age 27, female), 35876, (age 52, female).

### 2.4. Study Participants

The total study included 11 patients with distal esophageal cancer who were treated with neoadjuvant chemoradiation to 50.4 Gy with concurrent carboplatin and paclitaxel followed by esophagectomy. All patients gave written consent for blood collection and analysis. Table 1 contains the patients’ characteristics. Patients underwent fasting blood draw prior to the initiation of RT and 4–6 months following RT. The study was approved by the Georgetown University IRB (#2015–1320).

As this was a blinded study, to test the method of using methylation as a biomarker to detect organ damage, all patient data published in Burke et al., [31]. One patient was excluded in this manuscript due to limited sample collection.

### 2.5. Serum Collection

4 mL peripheral venous blood was drawn in serum tubes (BD Vacutainer), allowed to clot and spun down 1–2 hours later, in a swing bucket centrifuge at 1200 x g for 10 minutes. Serum was collected from the supernatant and frozen at −80 °C in 200 μL aliquots, until further analysis. A post-treatment sample was collected at the last day of radiation (5.5 weeks) and thus around 6 – 7 weeks after the pre-treatment sample. A third sample after recovery was collected 3 – 4 months after the last radiation dose.

### 2.6. DNA Isolation, Bisulfite Conversion and PCR

Serum was thawed on ice and cell-free circulating DNA (cfDNA) was isolated from 2 × 100 μL serum per patient sample using the DNA extractor SP Kit (Wako cat. # 296–60501), following the manufacturer’s protocol. The cfDNA was diluted in 15 μL of ultra-pure water and quantitated with the qubit fluorometric quantification (Thermo Fisher Scientific). Bisulfite conversion of 50 ng DNA per sample was performed using the EZ DNA Methylation-Gold Kit (Zymo cat. # D5005), following the manufacturer’s protocol. After purification and desulphonation, the DNA was diluted in 15 μL of ultra-pure water. PCR amplification was performed using the Platinum Taq DNA polymerase kit (Invitrogen cat. # 10966), in the following reaction: 2.5 μL of bisulfite converted DNA or genomic DNA, 39.4 μL of water, 5 μL PCR Buffer, 1.5 mM MgCl2, 0.2 μM dNTP, 0.2 μM primers, 2 Units of Platinum DNA Taq Polymerase. Reactions were incubated in the Epgradient Mastercycler (Eppendorf) thermal cycler. Cycling consisted of 2 min denaturation at 95 °C, followed by 38 cycles of: 30 seconds at 94 °C, 30 seconds at 55°C, then 30 seconds at 72 °C, and an infinite hold at 4 °C.

**Figure 2.**
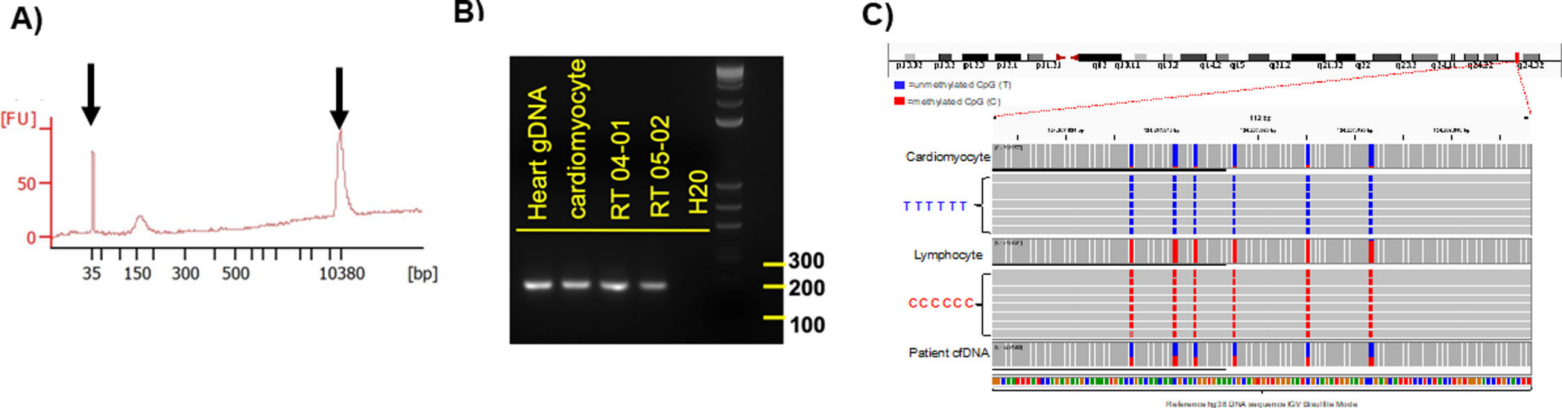
Detection of cardiomyocyte-specific methylated cfDNA. A) Bioanalyzer reading of cfDNA. Fragment size (~150bp) and cfDNA level in patient serum after DNA extraction. Peaks from size markers spiked in with the sample serve are a reference (arrows). B) Gel image of amplicons using primers adjacent to the FAM101A locus (see Methods) (Zemmour et al., 2018). DNA samples analyzed were genomic heart and cardiomyocyte DNA, patient RT04 and RT05 cfDNA from the current study, H_2_0 as a negative control. C) Bisulfite sequencing data from the FAM101A genomic locus (chr12:124,207,916–124,208,005) using cardiomyocyte and lymphocyte gDNA as well as patient cfDNA. The six CpGs in this locus are unmethylated in cardiomyocytes but methylated in lymphocytes. in bisulfite treated DNA from cardiomyocytes the six C’s read as Ts in contrast to lymphocytes where they read as C’s. The distinctive pattern can be used to identify cardiomyocyte-derived DNA molecules in patient serum relative to the total amount of DNA from all tissue sources.

Amplicons were examined by electrophoresis in 2% agarose gel with 1X TAE buffer, and visualized with xylene cyanol dye and ethidium bromide under UV light (see Fig. 2B). As a size marker the 1 kb plus DNA ladder (Invitrogen) was used. All PCR products that showed single bands at the expected size were evaluated by Sanger sequencing analysis. These selected primers were used for methylation PCR of the serum cfDNA samples. Sequences of selected primers used for cfDNA methylation analysis were based on Zemmour et al [19].

### 2.7. Determination of cfDNA Fragment Size

The fragment size of cfDNA from patient samples was determined for each sample with an Agilent High Sensitivity DNA chip, according to the manufacturer’s instructions. Agilent 2100 Bioanalyzer software was used and fragment size defined as the mode of the main peak in the electropherogram (see Figure 2A).

### 2.8. Sequencing Adapter Ligation

Illumina adapter overhangs were attached to the PCR amplicons using the Platinum Taq DNA polymerase Kit (Invitrogen cat. #10966), in the following reaction: 2.5 μL of purified PCR amplicons, 39.4 μL of water, 5 μL PCR Buffer, 1.5 mM MgCl2, 0.2 μM dNTP, 0.2 μM primers, 2 Units of Platinum DNA Taq Polymerase. Cycling consisted of 2 min denaturation at 95 °C, followed by 20 cycles of 30 seconds at 94 °C, 30 seconds at 56 °C, and 30 seconds at 72 °C. The samples were kept at 4 °C until further analysis.

Primer sequences for adapter ligation:

*Forward:* TCGTCGGCAGCGTCAGATGTGTATAAGAGACAGTATGGTTTGGTAATTTATT TAGAG

*Reverse:* GTCTCGTGGGCTCGGAGATGTGTATAAGAGACAGAAATACAAATCCCACAAATAAA

PCR Amplicons were examined by electrophoresis in 2% agarose gel as described above. PCR products were purified using the QIAquick PCR Purification Kit (Qiagen). Cleaned PCR products were eluted in 30 μL of water. The predicted size of the amplicon is 189bp.

### 2.9. Indexing and Library Preparation

Purified amplicon concentrations were quantitated with the QuantiFluor ONE dsDNA System (Promega cat. # E4870), following the manufacturer’s protocol. The amplicon was normalized to a concentration of 5 ng/μL in water and pooled per patient sample. Each amplicon pool was constructed into a dual indexed library using the Nextera XT Index Kit (Illumina cat. # FC-131–1001) and the 2x KAPA HiFi HotStart Ready Mix (KAPA Biosystems cat. # KK2602). The unique index sequence was added to each library sample through an 8-cycle PCR amplification procedure found in the 16S Metagenomic Sequencing Library Preparation user guide (Illumina Part # 15044223 Rev. B). Each sample was purified using AMPure XP beads (Beckman Coulter cat. # A63881) and as assessed using the Agilent DNA 1000 kit (Agilent Technologies cat. # 5067–1504) on the Bioanalyzer instrument. The libraries were normalized to 4 nM and pooled together to be sequenced on an Illumina MiSeq instrument.

### 2.10. Illumina MiSeq Deep Sequencing and Data Analysis

Before sequencing, an aliquot of the 4 nM library pool was denatured by incubating the aliquot with 0.2N NaOH for 5 minutes and then kept on ice. One percent of 12.5 pM PhiX Control V3 (Illumina) was spiked into the denatured library pool. Paired end 2×150 bp sequencing was performed on the MiSeq using the MiSeq Reagent Nano kit v2 (300 cycles) according to the manufacturer’s protocol (Illumina). All primary- and run-quality analyses were performed automatically on the MiSeq.

Amplicon sequenced reads were demultiplexed, aligned to the reference genome using a modified BWA-algorithm and analyzed through the BISulfite-seq CUI Toolkit (BISCUIT) tool suite for bisulfite-converted DNA methylation data (https://github.com/zhou-lab/biscuit). Reads were quality filtered to include only reads mapped to the primary alignment in proper pairs and with the correct insert size. Methylation sites, sequenced reference-mismatched Cs, were reviewed by manual visualization of alignments using IGV (Broad Institute) using the bisulfite CG mode for alignment coloring (Figure 2C). Methylation frequencies were quantified to estimate relative methylation rates at specific sites. We show the data as a fraction of total 6 CpGs divided by the total number of reads. Only DNA fragments that showed all 6 CpGs as unmethylated were counted. Abundance of cardiac-specific DNA is provided in genome equivalents. >35,000 reads were obtained for each of the amplicons generated from the cfDNAs. Between 35,000 and 74,000 reads were obtained for each of the amplicons generated from the cfDNAs isolated from serum samples. Only amplicons that showed all 6 CpGs as unmethylated were considered as a cardiomyocyte-specific signal. For detection of cardiomyocyte-specific cfDNA an amplicon with 6 CpGs was analyzed.

## 3. Results

### 3.1. Patient Characteristics

Eleven patients were enrolled between February 2016 and February 2018. The median age was 69 (range 37 – 80 years) and the majority of patients were male (82%). Patients had clinical stage T2 (n=2) and T3 (n=9) disease with clinical N0 (n=3) or N1 (n=8) nodal stage. The majority of patients had adenocarcinoma (n = 10) and one patient had squamous cell carcinoma (Table 1). Each patient was assigned a unique identifier from RT-01 to RT-11, with three different timepoints (pre-treatment (01), post-treatment (02) and recovery (03)). One patient was excluded in this manuscript due to limited sample collection.

### 3.2. Overview of the Amplicon Sequencing Method

An overview of the approach is shown in Figure 1. Cell-free DNA (cfDNA) was extracted from patient serum, and subsequently bisulfite converted. Bisulfite-converted DNA was then subjected to PCR amplification for a fragment that contains the tissue-specific methylation signature of interest. The DNA amplicons were subjected to deep sequencing to quantify reads with different methylation patterns.

**Figure 3.**
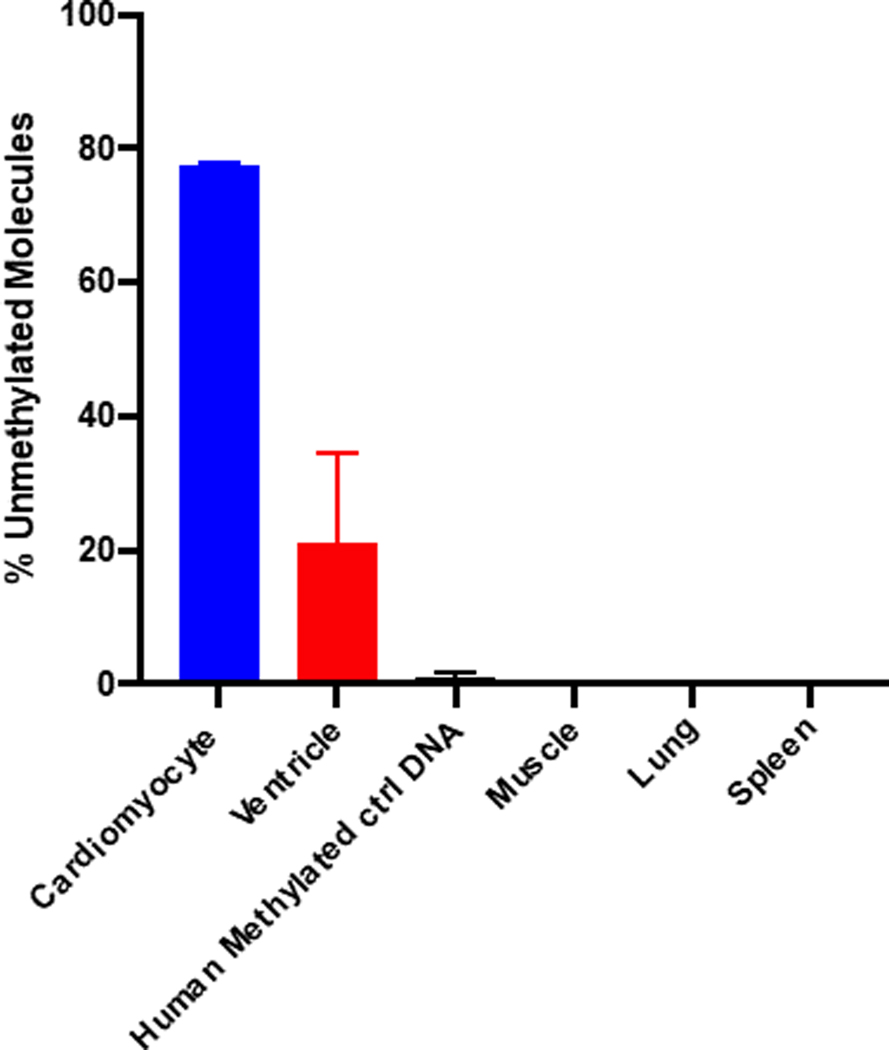
Cardiomyocyte-specificity of DNA methylation. Methylation levels of the FAM101A locus DNA in different human tissues, including cardiomyocyte, ventricle, skeletal muscle, lung and spleen plus human methylated control DNA. Mean ± SEM; n=2.

cfDNA extracted from serum had an average fragment size of ~150bp and a maximum of 200bp (Figure 2A). The FAM101A locus was selected for amplification because it contains a cardiac specific methylation patterns that is predicted to result in a 189 bp product from cfDNA after including the adapter sequences [19]. FAM101A was identified by Zemmour et al [19] by comparing methylomes of the human heart ventricle to the methylomes of 23 other human tissues. A cluster of cytosines adjacent to the FAM101A locus was selected. In this study, FAM101A was found to be cardiomyocyte specific, with 89% of the molecules fully unmethylated, in contrast to non-cardiac tissue where <0.2% of molecules were unmethylated, similar to the findings in Figure 3 [19]. Figure 2B shows a gel image with a set of PCR amplicons generated from genomic heart and cardiomyocyte DNA as a positive control, water as a negative control and two representative cfDNAs from patient serum. In the bisulfite sequencing analysis, only DNA fragments that showed all 6 CpGs as unmethylated were counted as a positive signal for cardiomyocyte-specific DNA. Figure 2C shows the sequencing reads from cardiomyocyte (positive control), lymphocyte (negative control) genomic DNA as well as examples of cell-free DNA from patient serum. Most cardiomyocyte DNA CpGs in this genomic region were unmethylated and read as Ts due to the bisulfite conversion whereas lymphocyte DNA was methylated and thus read as Cs (see Figure 1).

### 3.3. Verification of Cardiomyocyte Methylation Markers

**Figure 4.**
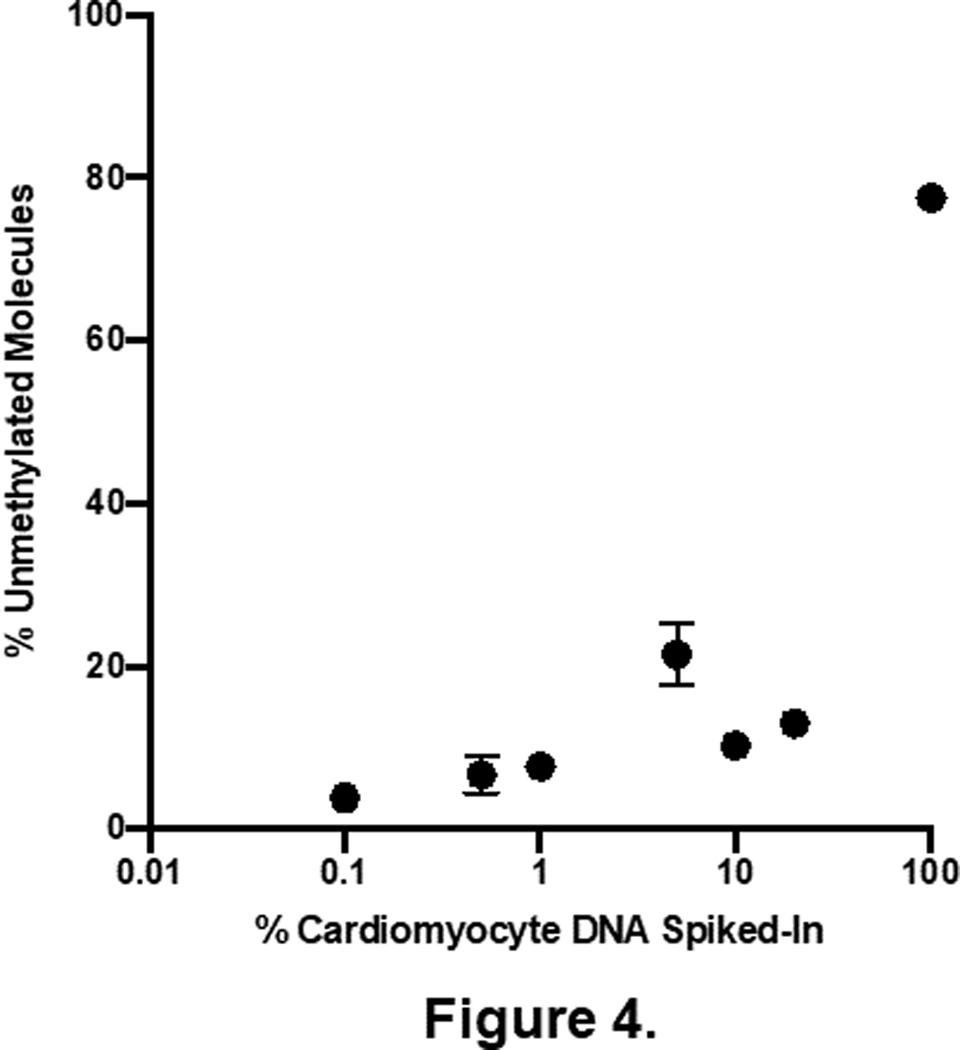
Detection of human cardiomyocyte DNA spiked into human buffy coat DNA. The percentage of fully unmethylated FAM101A locus molecules (in which all CpG sites were converted by bisulfite) was determined from amplicon sequencing. Mean ± SEM; n=3. The buffy coat DNA provides the background reading and will register as methylated sites in this locus.

We applied the amplicon sequencing method of the FAM101A locus to commercially available cardiomyocyte genomic DNA. We found 78% of the molecules were fully unmethylated at the FAM101A locus (Figure 3), which is similar to the finding of Zemmour et al [19] (89%). Cardiac ventricle DNA contained 20% of fully unmethylated molecules indicative of the presence of cell types other than cardiomyocytes in the tissue, and non-cardiac tissue such as muscle, spleen and lung showed <0.2% of molecules as unmethylated (Figure 3). As a control, we used human methylated DNA and found <0.9% of these molecules as unmethylated (Figure 3). It is noteworthy that the conversion rate of unmethylated C’s by bisulfite treatment of DNA was 99%. Spike-in experiments were performed to assess the sensitivity of the assay by mixing human cardiomyocyte DNA and human buffy coat DNA in a serial dilution from 0.1% to 100% cardiomyocyte DNA. The amount of cardiomyocyte DNA in the spike-in experiment was measured using PCR amplification and subsequent deep sequencing. We found detectable cardiomyocyte DNA spiked into human buffy coat as low as 0.1% of total DNA (Figure 4). In composite, these results indicate that the amplicon sequencing of the indicated CpG island in the FAM101A locus detects cardiomyocyte-specific DNA.

### 3.4. Circulating Cell Free DNA Analysis

Thoracic radiation therapy has been associated with the development of early and late cardiac toxicity. Our study included 10 patients with distal esophageal cancer that were treated with neoadjuvant chemoradiation to 50.4 Gy with concurrent carboplatin and paclitaxel followed by esophagectomy. Five patients (RT-02, 05, 06, 09, and 10) had pre-existing cardiac pathologies and three patients developed structural and functional clinical cardiotoxicity on follow up cardiac MRI scans within 6 months of chemoradiation. We analyzed serum samples drawn pre- and post-radiation treatment and after recovery to measure cardiomyocyte-specific cfDNA as an indicator of cardiac damage in these patients. Established circulating biomarkers of cardiac (BNP and Troponin-I) or systemic response to the radiation treatment (CRP) were also obtained (Supplementary Figure 2). Cardiomyocyte-specific methylated DNA was quantified relative to non-specific cfDNA present in the FAM101A amplicon. We hypothesize that the method is potentially more sensitive than current clinical tests in indicating cardiomyocyte turnover since BNP, Troponins, LVDEV and CRP in these patients were mostly normal.

**Figure 5.**
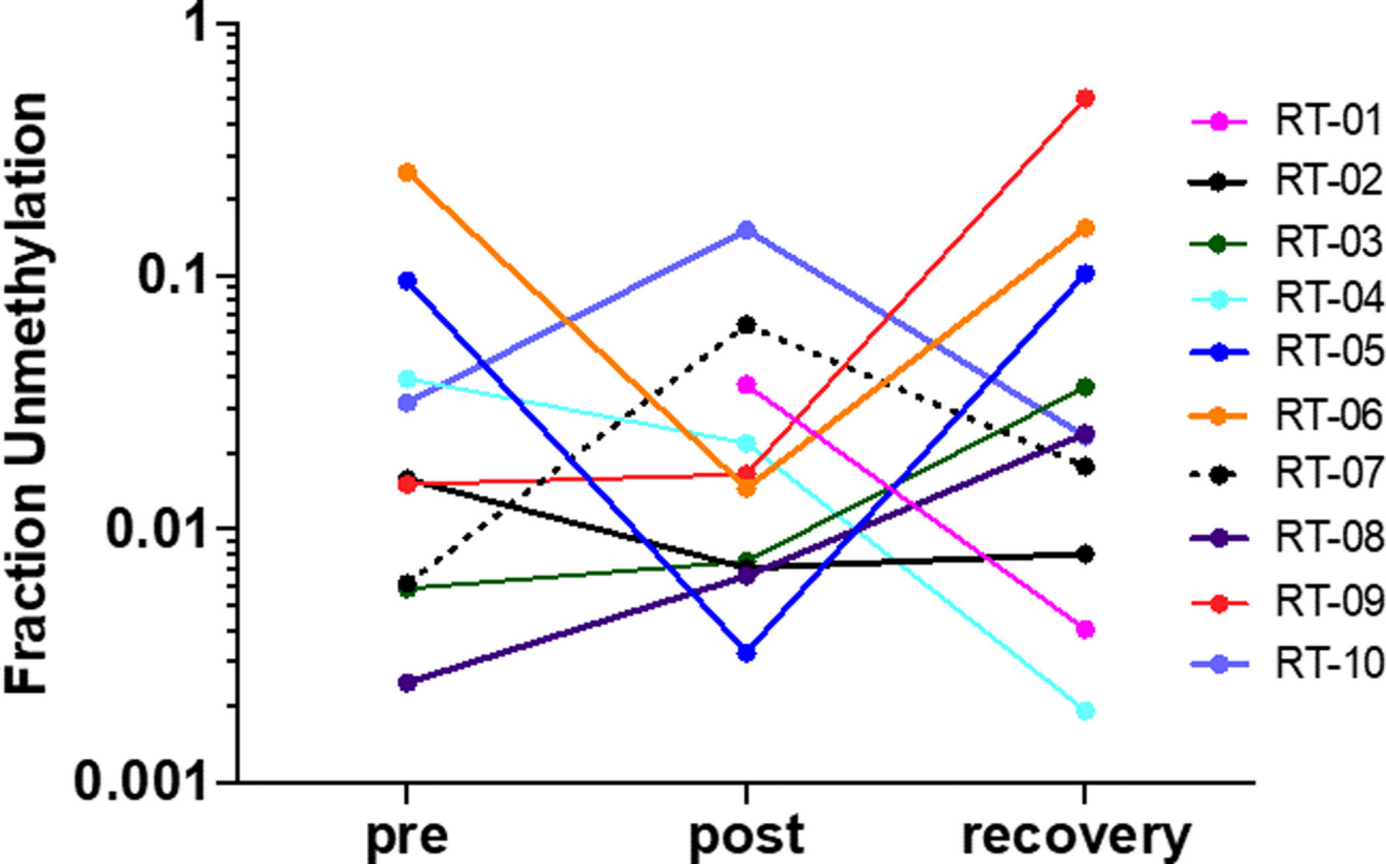
Cardiomyocyte specific methylated cell-free DNA in serum samples from patients with esophageal cancer pre- and post-radiation treatment and after recovery. Data from 10 patients are shown as fraction of unmethylated cfDNA calculated as the ratio of unmethylated DNA of all six contiguous C’s in the locus / methylated DNA. The grey box depicts the limit of detection in serum samples.

Samples with the highest levels of cardiomyocyte-specific cfDNA are from RT-06 at the pre-treatment timepoint and from RT-09 after recovery (Figure 5). It is noteworthy that RT-06 had preexisting cardiac damage and RT-09 had a pathologically elevated BNP for all time points corroborating cardiac pathology even though Troponin-I was in the normal range (see Supplementary Figure 2). Before initiation of radiation treatment six patients showed cardiomyocyte-specific cfDNA levels above the limit of detection (grey box in Fig. 5) possibly due to cardiac co-morbidity of damage from the primary disease. Interestingly, before initiation of treatment cfDNA levels in three patients (RT-03, −07, −08) with cfDNAs below the detection limit increased into the detectable range. Overall, the median cardiomyocyte-specific cfDNA levels in this cohort did not change significantly during treatment (0.018 – 0.020). We speculate that patient-specific cardiac damage or cardiac remodeling as well as disease control due to the treatment may be revealed by these changes.

Finally, to assess reproducibility across sequencing runs, we analyzed two separate complete preparations from DNA extraction to quantitation of cardiomyocyte-specific cfDNA amplicons. Repeated analysis of patient samples RT-04–01 and RT-05–02 (Supplementary Figure 1) showed similar readouts for the independent runs, suggesting that the method is reproducible and stable.

## 4. Discussion

This is the first study in which a cardiomyocyte cfDNA methylation marker was used to detect cardiac damage as a result of chemotherapy and radiation treatment in cancer patients. In this study, we established and evaluated the analysis of cardiomyocyte-specific, cfDNA as a biomarker of detecting and monitoring cardiotoxicity in cancer patients. The identification of tissue-specific, cfDNA relies on DNA methylation patterns that are highly cell-type specific. In order to detect cardiomyocyte-derived cfDNA in the circulation, we designed an amplicon sequencing method whereby first DNA was extracted from serum, then DNA was bisulfite converted, the gold standard method to distinguish between methylated and non-methylated CpGs (Figure 1). We quantitated the frequency of methylated cytosines by bisulfite sequencing of DNA amplicons containing the genomic region of interest (Figure 1 & 2). The sequence-based analysis used here increases the specificity of detection because we counted only those DNA fragments as cardiomyocyte-specific that showed a homogeneous lack of methylation at all six CpGs contained in the tissue-specific amplicon selected (Figure 2C). This approach reduces non-specific signals from random methylation of individual CpGs in the DNA fragment of interest [15]. The sequencing method was quality-controlled for distribution of sequencing quality and error rate distribution. Only samples that passed these steps were included. Cardiomyocyte-specificity was established using genomic DNA from human cardiomyocytes and from heart ventricle tissues. A higher percentage of DNA molecules with the specific methylation pattern was detected in human cardiomyocyte gDNA relative to ventricle tissue DNA. This difference is due to DNA from other cell types present in the ventricle sample. Both of these genomic DNA samples showed a higher percentage of unmethylated molecules than genomic DNA from human methylated DNA, skeletal muscle, lung and spleen (Figure 3). The data show that this marker can be used to detect cardiomyocyte specific DNA.

In this pilot study the sequencing depth was 35,000 to 74,000 read-pairs for the cfDNA samples which provides a very sensitive read-out to detect low abundance of differentially methylated amplicons. The combination of this deep sequencing of >35,000 reads and the requirement of homogeneous non-methylation of all six cytosines in the selected amplicon results in an exquisitely sensitive and specific assay to detect cell death even from normal cardiac cell turnover under physiologic conditions. The abundance of differently methylated cfDNAs in the circulation is an indicator of the steady-state turnover of different cell populations. Changes in cell type-specific cfDNAs indicate altered rates of cell death in a given cell population (see Introduction; [17]). Thus, the definition of a normal range is challenging because cell turnover can be impacted by a range of physiologic differences such as age, sex, body weight, ethnicity but also by co-morbidities such as hypertension and metabolic disease or by medications that target inflammation and thus cell turnover. In therapeutic studies such as the one here, serial analyses of samples collected before and after treatment is the best approach to avoid biases due to person-to-person variations due to the multitude of conditions. Also, as more data are generated with a multitude of physiologic and pathologic situations, a better understanding of the impact of those variables will emerge. In the majority of patients in this study cardiac cfDNA was detectable above the baseline before radiation therapy onset which could be due to local reaction to the invasive cancer or pre-existing cardiac co-morbidity picked up by this sensitive readout.

Some weaknesses in this pilot study are due to confounding factors that include the variable extent of the disease across the patient cohort and different pre-existing cardiac co-morbidity at the time of treatment and sample collection. Also, we did not find a significant relationship between changes in cardiomyocyte cfDNA abundance and other established cardiac damage parameters such as Troponin-1 (Suppl Fig. 2). This may be due to different residence time in the circulation of Troponin-1 versus the relatively short cfDNA half-life leading to different steady state levels. In studies with defined timing of cardiac intervention (i.e. PCI) Troponin-1 showed a distinctly longer residence time than cardiac cfDNA [19]. An alternative approach to detect adverse cardiac effects applies pattern analyses of metabolites in the circulation that is indicative of tissue specific damages. Feasibility of this approach was shown recently [32]. Metabolomic changes in cardiac tissues and the circulation after radiation treatment were established in an animal model and the patterns were then used to identify at risk patients in the cohort also described here.

From the data presented we conclude that we can detect cardiomyocyte specific cfDNA in the circulation with high sensitivity, potentially supporting monitoring in patients undergoing treatments with known cardiotoxic adverse effects.

**Publisher’s Note:** MDPI stays neutral with regard to jurisdictional claims in published maps and institutional affiliations.

## Supplementary Material

Suppl. Figure 1

Suppl.Figure 2

Suppl. Materials

## Figures and Tables

**Table 1.** Patient characteristics in the study.

Age (years)	
Median	69
Range	37–80
**Sex(%)**	
Male	9 (82%)
Female	2 (18%)
**Tumortype(%)**	
Adenocarcinoma	10 (91%)
Squamous cell carcinoma	1 (9%)
**Clinical N stage (%)**	
cT2	2(18%)
cT3	9 (82%)
**Stage group(%)**	
IIA	3 (27%)
III	8 (73%)
**Heart Comorbidities**	
Coronary Artery Disease, Hypertension and/ orHyperlipidemia	6 (60%)
