# Supplementary figures and images for: Cardiomyocyte-specific circulating cell-free methylated DNA in esophageal cancer patients treated with chemoradiation
Source: Gastrointest Disord (Basel). Author manuscript; Available in PMC 2022 May 6. (PMC9074856; doi:10.3390/gidisord3030011)

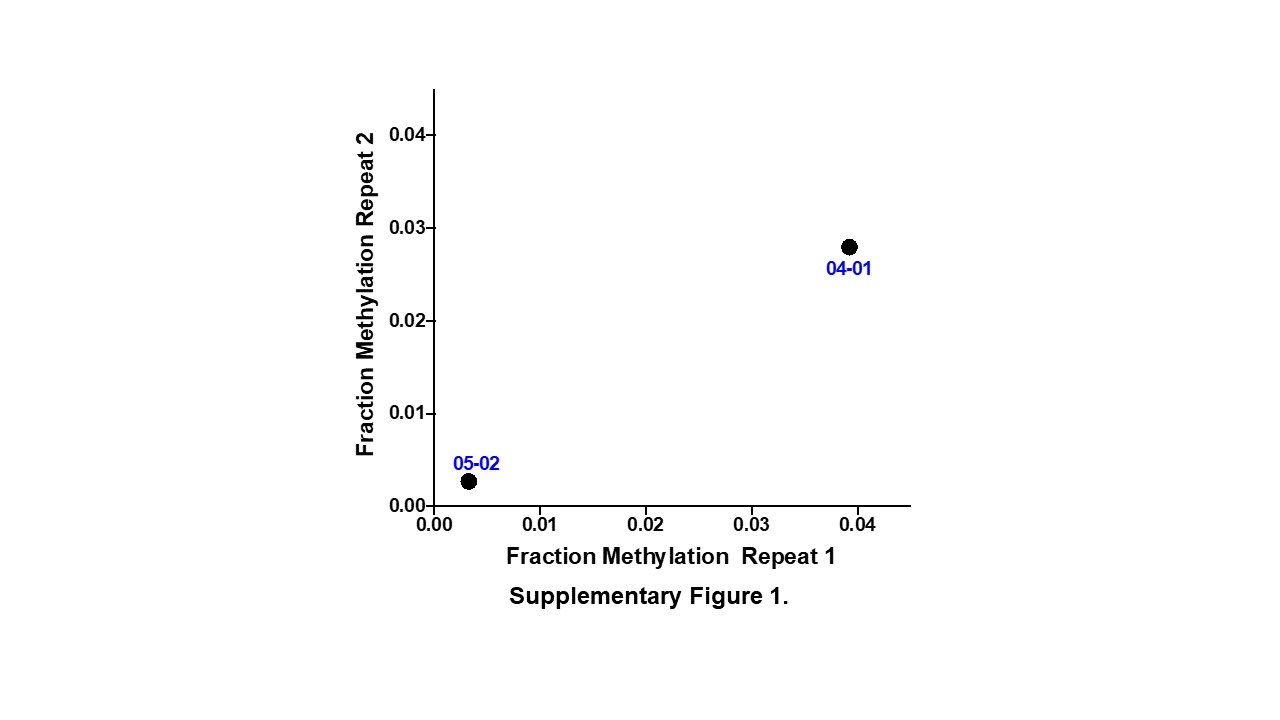

Supplement: Suppl. Figure 1 [file NIHMS1733460-supplement-Suppl__Figure_1.png]

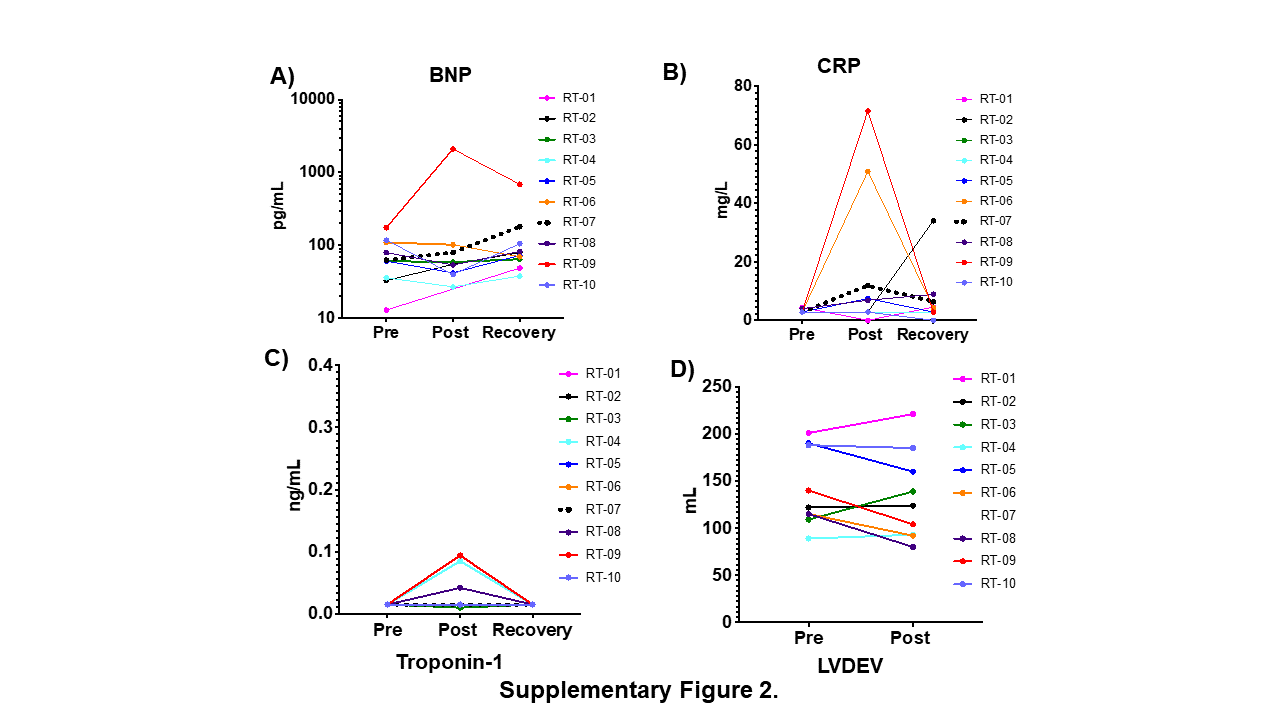

Supplement: Suppl.Figure 2 [file NIHMS1733460-supplement-Suppl_Figure_2.png]
