# Supplementary material for: Cardiomyocyte-specific circulating cell-free methylated DNA in esophageal cancer patients treated with chemoradiation
Source: Gastrointest Disord (Basel). Author manuscript; Available in PMC 2022 May 6. (PMC9074856; doi:10.3390/gidisord3030011)
Supplement: Suppl. Materials [file NIHMS1733460-supplement-Suppl__Materials.pdf]

Supplementary Materials

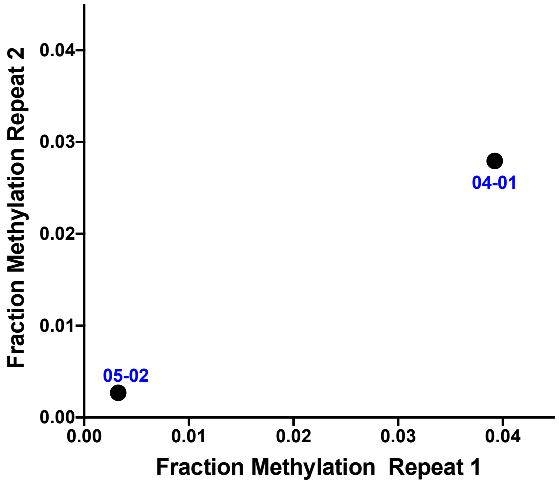

Figure S1. Repeat measurements of cardiomyocyte-specific methylated cfDNA in patient serum. Independent repeat measurements (from isolation to sequencing) of samples from patients RT05-02 and RT04-01 [Ma1](blue). Data are from separate serum DNA extractions and sequencing analysis. $n = 2$ per data point.

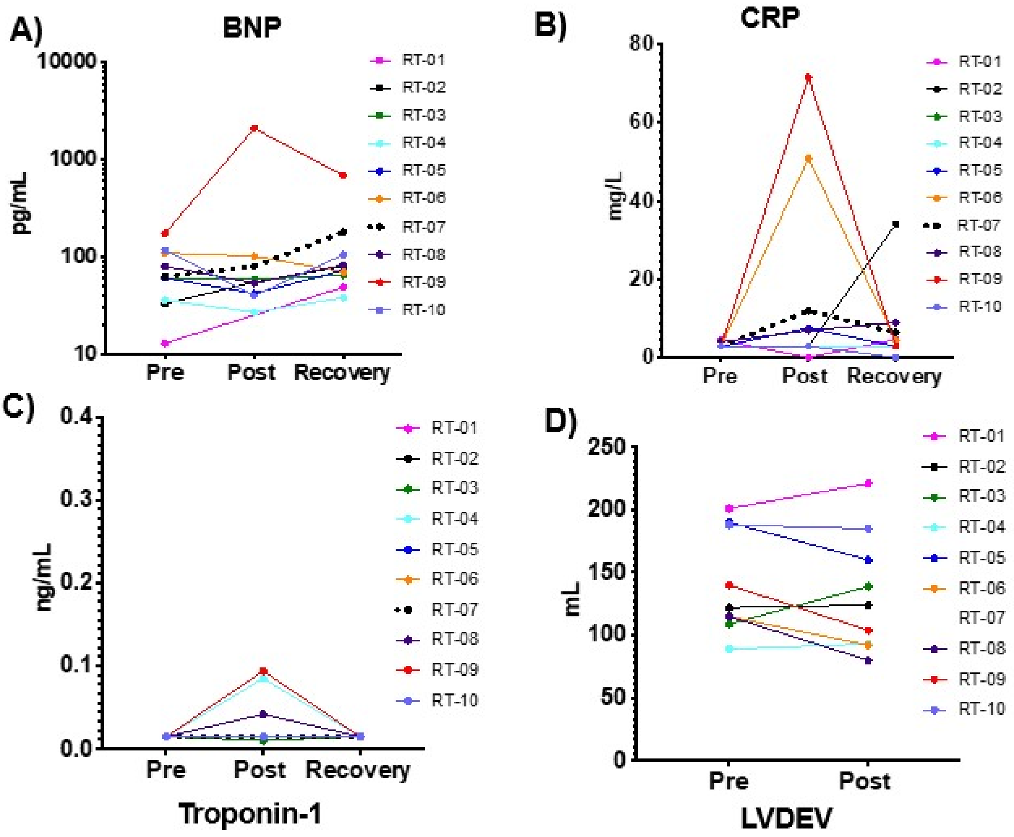

Figure S2. Serum and cardiac function markers (**A**) Clinical parameters patients BNP values, for 3 time points before after and at recovery, normal BNP values are less than 100 pg/mL shown in grey (**B**) Clinical parameters patients CRP values, for 3 time points before after and at recovery, normal

CRP values are less than 3 mg/L shown in grey (**C**) Clinical parameters patients troponin-I values, for 3 time points before after and at recovery, normal range 0–0.4ng/mL shown in grey (**D**) Cardiac function LVDEV values pre and post recovery. Not shown in the figure attached to the email.
